# Experiences and perceptions of everyday decision-making in the lives of adults with intellectual disabilities, their care partners and direct care support workers

**DOI:** 10.1177/17446295231189020

**Published:** 2023-07-12

**Authors:** Hannah Casey, Áine Trayer, Deirdre Desmond, Laura Coffey

**Affiliations:** Department of Psychology, 91089 Maynooth University, Maynooth, Ireland; Assisting Living and Learning Institute, 91089 Maynooth University, Maynooth, Ireland; Department of Psychology, 91089 Maynooth University, Maynooth, Ireland; Department of Psychology, 91089 Maynooth University, Maynooth, Ireland; Assisting Living and Learning Institute, 91089 Maynooth University, Maynooth, Ireland; Department of Psychology, 91089 Maynooth University, Maynooth, Ireland; Assisting Living and Learning Institute, 91089 Maynooth University, Maynooth, Ireland

**Keywords:** everyday decisions, intellectual disability, supported decision-making, self-determination, autonomy, systematic review

## Abstract

Decisional support is important to people with intellectual disabilities. This review explores: i) how everyday decision-making is perceived and/or experienced by adults with intellectual disability, their care partners and direct care support workers (DCSWs); ii) techniques/ approaches used to support everyday decision-making; and iii) barriers/facilitators encountered. PRISMA systematic review methodology using PsycInfo, PubMED, Web of Science, CINAHL and Scopus. Eighty-one papers were included [qualitative (*n* = 69), quantitative (*n* = 7), mixed methods (*n* = 5)] was used. Adults with intellectual disability reported wanting to makedecisions and needing support. Care partner support was affected by concerns about safety and decisional capacity. DCSWs reported difficulty balancing client decisions and care partner concerns when providing support. Supported Decision-Making (SDM) was identified as a key method of support. Barriers and facilitators were interconnected and impacted by stressors. This topic is under-researched and ill-defined. Supported decision-making is an increasingly popular approach whose application requires further exploration.

## Introduction

There is growing recognition of the need for effective supports to enable people with intellectual disabilities to be active participants in decision-making across life domains, with individualised supports matched to the scale of the decisions and the social and personal contexts that impact decision-making (Blanck and Martinis, 2018; Shogren et al., 2017). Supported decision-making (SDM) is fast gaining traction as an effective means for increasing self-determination by empowering people to make decisions about their lives to the greatest extent possible (Davidson et al., 2015; William and Porter, 2017). SDM has many definitions, but fundamentally rests on the premise that the person who requires support chooses someone to assist them in making decisions, with the final decision resting with the supported person (Center for Public Representation, 2021; Inclusion Ireland, 2021).

SDM is recognised in Article 12 of the United Nations Convention for the Rights of Persons with Disabilities (UNCRPD), which stipulates that governments must take appropriate action to provide people with access to the supports they need and want to make their own life decisions (United Nations, 2006). In their review of the literature, Davidson and colleagues (2015) summarised succinctly the necessity for this Article by outlining that while a person’s decisional capacity (ability to make decisions) may fluctuate, this should not mean their legal capacity (ability to exercise legal decisions) is removed, and recognised SDM as an important tool to facilitate this. This does not always occur in practice, however, particularly in the case of older adults or those with more severe intellectual disabilities, who are more likely to have substitute decision-making employed on their behalf (Watson, 2016). Everyday decisions (i.e., where to go, what to eat, whom to visit) form the bulk of decisional events for adults with intellectual disabilities (Bigby et al., 2015b). It is important to examine how people with intellectual disabilities can be best supported to make them. Previous reviews of the literature on decisional support among people with intellectual disabilities have focused on critical decisions such as end-of-life care or vital medical care (Noorlandt et al., 2020; Penzenstadler et al., 2020; Shogren et al., 2017). Reviews examining everyday decision-making have focused on people with TBI (Noorlandt et al., 2020) and dementia (Kohn and Blumenthal, 2014) rather than intellectual disability; or have recruited diverse samples (e.g. Bigby et al., 2015b; Penzenstadler et al., 2020; Shogren et al., 2017). The aims of this systematic review are to investigate i) how everyday decision-making is perceived and/or experienced by adults with intellectual disability, their care partners (a term used to include both familial and non familial support not carried out by a paid professional) (Daly et al., 2018) and direct care support workers (DCSWs, i.e., professional staff working directly with adults with intellectual disability); ii) what techniques/approaches to decisional support are used in this context; and iii) barriers/facilitators to effective decisional support encountered. For the purposes of this review, an everyday decision is one which any person may make any day throughout their life as a natural consequence of being an adult, outside of a critical life event such as severe illness or end of life measures. We have used the Everyday Decision-Making project carried out by the University of Birmingham as a guide for this (Harding and Tascioglu, 2017).

## Method

This review followed the Preferred Reporting Items for Systematic Reviews and Meta-Analysis (PRISMA) guidelines (Page et al., 2021) and was registered with PROSPERO (CRD4202170417; https://www.crd.york.ac.uk/prospero/display_record.php?RecordID=170417).

### Search strategy

Electronic searches of CINAHL, PsycInfo, PubMed, Scopus and Web of Science were performed in May 2020 and updated in February 2022. Searches were limited to articles published in English since January 2006 to anchor the review in the landmark publication of the UNCRPD. Search strings of controlled vocabulary and/or free text search terms were developed for each database using search strategies of relevant reviews (Bigby et al., 2015b; Daly et al., 2018) for guidance, and reflected the varying terminology used to indicate intellectual disability over time and across countries (Table 1).

**Table 1.** Summary of Search Strings Used for Included Databases.

Database	Search String
PubMed	((“Intellectual Disability”[Mesh]) AND “Decision Making”[Mesh] OR “ Decision Making, shared” [Mesh] + year 2000 – 2020
Scopus	“intellectual* disab*” OR “intellectual* handicap*” OR “intellectual* impair*” OR “intellectual* disorder*” OR “development* disab*” OR “mental* handicap*” OR “mental* disab*” OR “mental* retard*” OR “down* syndrome*” AND “decision making”
Web of Science	(TS=(“intellectual* disab*” OR “intellectual* handicap*” OR “intellectual* impair*” OR “intellectual* disorder*” OR “development* disab*” OR “mental* handicap*” OR “mental* disab*” OR “mental* retard*” OR “down* syndrome*”) AND TS=(“decision making”)) *AND *LANGUAGE: (English). From 2000-2020
PsycInfo	intellectual* disab* OR intellectual* handicap* OR intellectual* impair* OR intellectual* disorder* OR development* disab* OR mental* handicap* OR mental* disab* OR mental* retard* OR down* syndrome* AND decision making (LIMIT: English, 2000-2020)
CINAHL	intellectual* disab* OR intellectual* handicap* OR intellectual* impair* OR intellectual* disorder* OR development* disab* OR mental* handicap* OR mental* disab* OR mental* retard* OR down* syndrome* AND decision making (LIMIT: English, 2000-2020)

The word “decision” is often used interchangeably with “choice” in the literature. In our refinement of our search terms and inclusion criteria, we noted some researchers have attempted to draw a distinction between the two by categorising smaller, less significant responses as choices, while labelling more significant ones as decisions (Harris, 2003). However, Brown and Brown (2009), when outlining their five-step approach to choice making, assert that ultimately, both allude to the right of a person to select and respond to any such choice or decision on their own terms. Therefore, we chose not to exclude papers on the basis of which term was used.

### Article selection criteria

Papers were eligible if they included adults (≥18 years) with intellectual disability, their care partners and/or DCSWs. We chose the term ‘care partner’ to describe non-professional decisional supporters, including non-relatives (Daly et al., 2018). Papers including care partners and/or DCSWs only were ineligible if they were not directly involved in supporting everyday decision-making. Papers reporting primary data on one or more of the following topics were included: i) experiences/perceptions regarding the provision or receipt of support for everyday decision-making; ii) approaches/techniques for supporting everyday decision-making; iii) barriers/facilitators of effective support in everyday decision-making. Articles specific to advance care planning, hospital directives or end-of-life care were excluded.

Search results were uploaded to Mendeley citation manager (Reiswig, 2010), where duplicates were removed, then transferred to Rayyan (Ouzzani et al., 2016) for screening. Two reviewers (HC and AT/LC) screened the titles/abstracts independently for eligibility, followed by the full-texts of those deemed potentially eligible. Both screening stages were followed by a joint conflict review, then brought to a third reviewer (DD) for a final decision if required (Table 2).

**Table 2.** Inclusion Exclusion Criteria for Systematic Review.

	Inclusion Criteria	Exclusion Criteria
Publication language	English	Languages other than English
Publication date	2006–2020, updated to include 2020-2022	Prior to 2006
Target population	Adults aged 18 and over with intellectual disabilities; care partners of adults with intellectual disabilities; direct care support workers of adults with intellectual disabilities	Participants with cognitive impairments other than intellectual disabilities; direct care support workers who are not involved in decisional support for adults with intellectual disabilities
Study focus	How adults with intellectual disabilities, their care partners and/or direct care support workers perceive/experience supported decision making in everyday life; practical, applied techniques and approaches to supported decision-making; barriers/facilitators of supported decision making in everyday life; Conflict or complications in supported decision making experienced by care partners or direct care support workers.	Studies focusing on methods of decision-making that do not include the adult with intellectual disabilities themselves
		Studies focusing exclusively on advance care planning, end of life care, or terminal care decisions; Decision-making by an external agent such as a solicitor or other legal professional with no inclusion of the person with intellectual disabilities
Study type	Primary data, all research designs (qualitative, quantitative, mixed methods)	Secondary data
		Non-peer reviewed publications (opinion pieces, editorials, conference abstracts, magazine articles, letters to the editor etc.)
	Peer reviewed publications	Systematic reviews

### Data extraction

Data extraction for the initial search was completed by AT and by HC for the updated search. Data extracted from each article included: author(s) name, publication year, country, study design, aim(s) and research questions, participants and sample size, data collection method, response rate, method(s) of data analysis, and findings (Supplementary Table 2).

### Quality assessment

Included articles were critically appraised by HC and LC using the Mixed Methods Appraisal Tool (MMAT: Pace et al., 2012), with DD consulted for any unresolved conflicts. The MMAT is designed to appraise the quality of qualitative, quantitative and mixed methods publications using a single tool. Papers are screened for relevance using two questions, then further assessed using five design-specific questions. The quality review was conducted to aid readers’ critical consideration of the included articles and the credibility of their findings, no papers were excluded on this basis (Supplementary Table 1).

### Data synthesis

Content analysis was used to synthesise the data. This is a flexible methodology with versatile applications (Drisko and Maschi, 2016; Fingfeld-Connet, 2014; White and Marsh, 2006), and was chosen for the current review given the broad nature of the research questions, and diversity of research methods and analytic techniques employed. Both deductive and inductive approaches were used; papers’ contents were categorised according to their relevance to the review’s three aims, while also allowing the data to indicate relevant subthemes. HC conducted the content analysis in a series of steps, guided by Evans and Fitzgerald's (2002) approach. Firstly, all papers were read and reread to establish familiarity with their outcomes and objectives. Papers were initially categorised according to participant group. Where more than one participant group was included, data from each group were analysed separately. Papers were then clustered by topic according to the review’s three aims.

### Study characteristics

**Figure 1.**
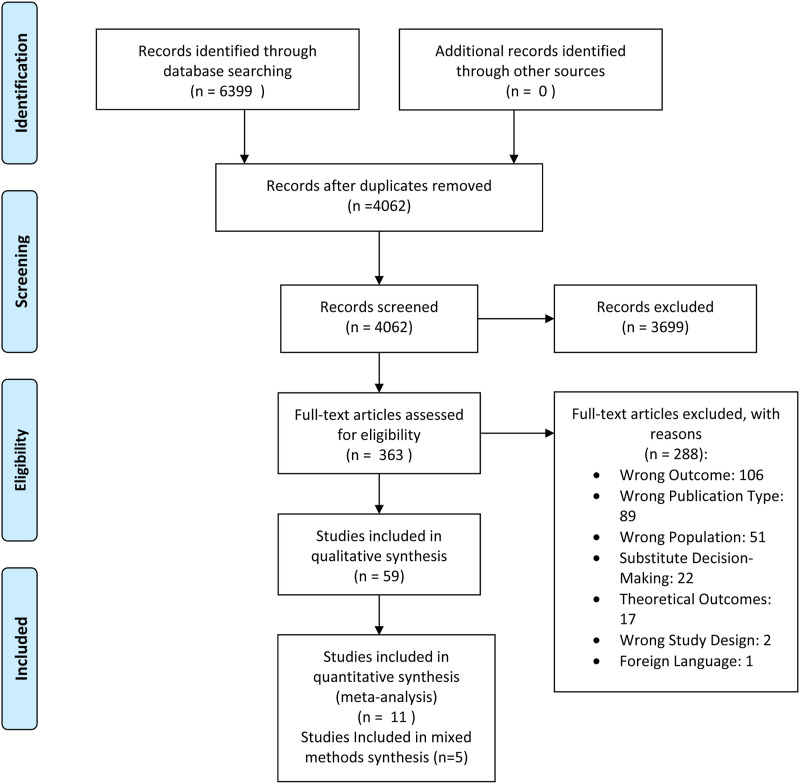
PRISMA 2009 flow diagram. *From:* Moher D, Liberati A, Tetzlaff J, Altman DG, The PRISMA Group (2009). *P*referred *R*eporting *I*tems for *S*ystematic Reviews and *M*eta-*A*nalyses: The PRISMA Statement. PLoS Med 6(7): e1000097. doi: 10.1371/journal.pmed1000097. For more information, visit https://www.prisma-statement.org.

Of the 4915 papers (excluding duplicates) identified, 81 papers reporting the findings of 76 studies were included (Figure 1). Articles originated from the UK (n = 31), USA (n = 11), Australia (n = 12), Ireland (n = 7), Spain (n = 4), Sweden (n = 3), Israel (n = 2), New Zealand (n = 2) Malta (n = 2), Norway (n = 2), Canada (n = 2), Belgium (n = 1), Iceland (n = 1), and China (n = 1). Studies used qualitative (n = 69), quantitative (n = 7), and mixed methods (n = 5) designs. Supplementary Table 2 provides a summary of included papers’ characteristics .

## Results

Study findings were considered in terms of the review’s three aims. 1) experiences/perceptions of everyday support for decision-making; 2) techniques/approaches to everyday support for decision-making employed; and 3) barriers/facilitators of effective support for decision-making. Where relevant, quotes or extracts from included studies are provided in Supplementary Table 3. A select number of studies are highlighted as examples within this section.

### Experiences and perceptions of support in everyday decision-making

Of the 33 papers which explored experiences and perceptions of support for everyday decision-making, 25 included adults with intellectual disabilities, 9 included care partners, and 7 included DCSWs.

#### People with Intellectual Disabilities

Participants with intellectual disability reported they appreciated the support of carers during decision-making, but sometimes felt underestimated, or that their preferences were not taken seriously (Bigby et al., 2019; Brotherton et al., 2020; Carey et al., 2021; Collings et al., 2019; Curryer et al., 2018; Lukas et al., 2018). External pressure to make certain choices, or having decisions made for them (Nonnemacher and Bambara, 2008; Werner and Chabany, 2016), led to negative perceptions or experiences. Proximity to care partners (Burke et al., 2019; Curryer et al., 2018) and participation in self-advocacy programmes or groups (Deguara et al., 2012; Gilmartin and Slevin, 2010; McCausland et al., 2018) were associated with positive experiences. Gilmartin and Slevin (2010) reported that adults with intellectual disability in a participating advocacy group found their membership empowering and gave them more confidence in expressing their preferences during Person Centred Planning (PCP) meetings. Similarly, Espiner and Hartnett. (2012) found that PCP meetings in which both care partners and DCSWs were present allowed adults with intellectual disabilities to be more open about their goals and plans when the meeting facilitator had been trained in inclusive meeting techniques. Wong and colleagues (2021) observed that in discussions about decisions to be made, the magnitude of the decision and the potential safety of the person affected which group had more influence in the decisional process (Wong and Chow, 2021).

Less positive experiences were also reported, such as being hampered by a requirement to have staff member accompaniment in all activities (Larkin et al., 2018), feeling pressurised by others to make certain decisions, or not being permitted to make decisions at all (Nonnemacher and Bambara, 2011; Werner and Chabany, 2016), which were associated with great frustration and unhappiness. In a paper written by members of an advocacy group for adults with intellectual disability based in Malta, Deguara and colleagues (2012) reported that they felt they were encouraged to participate in activities that carers thought they would like, rather than activities they themselves had chosen. In Brotherton and colleagues’ (2020) exploration of why older adults with intellectual disabilities chose to retire, many participants disclosed that they retired because carers asked them to do so. Carey and colleagues (2021) reported that adults with intellectual disabilities who had opportunities to make their own decisions, or to adapt their decisions to environmental factors, were happier than those who did not, or could not do this.

People with intellectual disabilities felt that at times, carers assumed more authority than appropriate, hindering their ability to assert themselves (Dowling et al., 2019; Goldsmith et al., 2013; Timmons et al., 2011). Consent was assumed and not explicitly sought in medical settings (Goldsmith et al., 2013; Sheehan et al., 2019), and decisions made in matters such as employment were often heavily influenced by supporters (Timmons et al., 2011). Antaki and colleagues (2006, 2008, 2009) documented how DCSWs often ignored choices or suggestions put forward by their clients and instead used conversational techniques such as directive guiding, piloting them to a specific answer or offering choice while already pursuing a course of action.

#### Care Partners

Findings indicated that care partners felt they represented an important bridge between the adult with intellectual disability and DCSWs because of their familiarity with the personality, preferences, and communication methods of the person (Bigby et al., 2015a; Burke et al., 2019). Their acceptance of the person’s choices and decisions appeared to be informed by their own personal values, and they often attempted to redirect the person if they felt their decision was inappropriate or unsuitable. This was grounded in a conviction that they knew the person with intellectual disability best (Brady et al., 2019; Curryer et al., 2020; Mannan et al., 2011; Werner and Chabany, 2016) and a desire to protect them (Werner and Chabany, 2016).

However, care partners were often the person with intellectual disability’s strongest advocates in decision-making. In a study of decision-making among sibling dyads by Burke et al. (2019), siblings of adults with intellectual disability were often outspoken advocates and defended their sibling’s right to be included and respected. Similarly, Bigby and colleagues (2011) observed care partners challenging decisions made by support workers in relation to transitions of older adults with intellectual disability to geriatric care settings. Finally, in a 2018 survey of Irish adults with intellectual disability, McCausland and colleagues reported that those who lived at home had more opportunities to make their own decisions than those in residential care.

#### Direct Care Support Workers

Many papers reported that DCSWs saw themselves as having a uniquely difficult position, often struggling to reconcile client decisions with care partners who disapproved of them (e.g. Andre-Barron et al., 2008; Bigby et al., 2011, 2019). At times, particularly where clients had severe intellectual disability and were presumed incapable of making choices for themselves, support in decision-making was not offered (Bigby et al., 2009); instead, best interests decision-making approaches, informed by DCSWs’ assumptions and opinions, were evident (Bigby et al., 2009; Rogers et al., 2020).

DCSWs placed more emphasis on neutrality in support than care partners. They were aware of the influence they could have over the person’s decisions, especially if the person they were supporting was noticeably attached (Bigby et al., 2019). In addition, Bigby and colleagues (2011) reported that DCSWs were often limited by policy considerations or the physical health of the person they were supporting, and sometimes felt they had to override the person’s preferences for their safety (Davies et al., 2017). Rogers and colleagues (2020) reported that clinical psychologists who worked with adults with intellectual disabilities felt that a culture of presumed “incapacity” prevailed in their workplace, with many DCSWs inadvertently wanting to take a protective rather than empowering approach to support. These difficulties were further exacerbated by regulations, bureaucratic practices, staff shortages and time constraints (Bigby et al., 2011, 2019). However, DCSWs also expressed a clear desire to encourage and support decision-making and independence, and frequently relied on the input of care partners to enact this. In Bigby and colleagues’ 2015 paper on sibling involvement in care and decision-making, DCSWs reported working closely with siblings to interpret what the person might want to do if they could not communicate, and relying on them for insight into the person.

### Techniques and approaches to support in everyday decisions

Of the 30 papers reporting approaches and techniques used to support everyday decision-making for adults with intellectual disability, 11 focused on SDM and included all three stakeholder groups; 19 papers explored other techniques and approaches to supporting everyday decision-making, which were targeted towards one stakeholder group only.

#### Supported Decision-Making

Supported decision-making (SDM) was explicitly used or directly mentioned in 11 papers (Bigby et al., 2019, 2022a, 2022b; Brotherton et al 2020; Browning et al., 2021; Buhagiar and Azzopardi Lane, 2022; Carney et al., 2021; Devi et al., 2020: Ledger et al., 2016; Webb et al., 2020; Werner and Chabany, 2016). Four studies focused on exploring attitudes to SDM among DCSWs and care partners (Bigby et al., 2019; Ledger et al., 2016; Werner and Chabany, 2016); 2 focused on piloting and evaluation of training programmes to encourage the use of SDM (Bigby et al., 2022a; Buhagier and Azopardi Lane, 2021); 5 involved explorations of techniques employed by carers and people with intellectual disabilities when engaging in SDM (Carney et al., 2021; Bigby et al., 2022b; Browning et al., 2021; Devi et al., 2020; Webb et al., 2020). Three papers detailed the application of the LaTrobe Framework (Bigby et al., 2022a, 2022b; Carney et al., 2021), an Australian training programme for stakeholders to learn how to apply SDM in their daily lives.

#### Differences in views between care partners and DCSWs

Both groups asserted that knowing the person well and being familiar with their personality was of vital importance, but the applications of these beliefs differed. DCSWs favoured a more neutral approach compared to care partners, who believed their knowledge of the person meant they could determine what was best for them (Bigby et al., 2019; Werner and Chabany, 2016). Werner and Chabany’s 2016 paper explored the views of Israeli parents of adults with intellectual disabilities on SDM compared to guardianship. Parents thought SDM was unrealistic, as they did not believe their children could make their own decisions, while the adults with intellectual disabilities themselves were unsure about SDM but liked the idea of discussing options and being included. Similarly, Ledger and colleagues (2016) conducted a survey of parents and staff who reported using SDM to support the contraceptive choices of adults with intellectual disabilities. Although respondents reported supporting an adult with intellectual disabilities to make decisions on this topic, the adult themselves rarely had the final say. Inclusion in decision-making was influenced by the level of support the person required, and failure to consult them was attributed to lack of easy-read information, GP preference, and in the case of adults with high support needs, the need for medication to manage menstruation.

#### Views of Adults with Intellectual Disabilities

Webb and colleagues (2020) reported that people with intellectual disabilities felt it was important to be involved in decision-making in their own lives. Decision-making gave them a sense of confidence, but they also reported needing help at times due to previous poor decisions they felt they had made. Buhagier and colleagues (2021) echoed this in their reporting on their financial abuse training programme. They found that people with intellectual disabilities wanted to have the final say on how much financial support they received, but agreed that they needed help to manage money effectively, through the training programme as well as from supporters.

Devi and colleagues (2020) identified three levels of decisions that they observed in their ethnographic research, based on the perceived level of support the person required to make it: spontaneous decisions made every day, which needed very little support; mid-level decisions such as helping the person fill out a medical information form; and strategic decisions, which required more time and senior staff involvement. They concluded that this approach was in line with Article 12 of the CRPD, which stresses that the will and preferences of the person be respected in decision-making. Carney and colleagues (2021) also stressed the importance of Article 12 and argued that certain decision-making techniques adopted by parents when supporting decision-making were not as paternalistic in nature as they might appear. Framing the decision to highlight a particular option or narrowing the field of choice for the person were shown to increase the comfort of the supported person and prevented decisional overload.

#### The LaTrobe Framework

Three papers included were based on the LaTrobe Framework (Bigby et al., 2022a, 2022b; Carney et al., 2021) developed by Christine Bigby and colleagues to assist people with intellectual disabilities and their carers in carrying out SDM. The framework consists of seven steps: 1. knowing the person, 2. identifying and describing the decision, 3. understanding will and preferences, 4. refining the decision to take into account of constraints, 5. considering if a formal process is needed, 6. reaching the decision and associated decisions, 7. implementing the decision and seeking advocates if necessary (Bigby et al., 2022a). In a study by Bigby et al. (2022a), parents of adults with intellectual disabilities who completed a training programme using this framework participated in repeated interviews to establish if the programme led to a change in approach in decisional support. After completing the programme, parents reported being more aware of how to use a structured approach to SDM, mitigate unconscious influence of the person, and broaden the circle of support. This contrasted with the findings of an earlier paper on parental strategies of decisional support, where parents sought to ensure they made the “right” decision out of concern and protectiveness (Bigby et al., 2022b) and controlled the types or extent of the decisions made or used their influence to guide to the outcome. They also reported using their influence to encourage participation in activities they thought would broaden their horizons or increase their level of healthy risk, however.

Browning and colleagues’ 2021 paper exploring Canadian approaches to SDM also discussed encouraging healthy risk using knowledge of the person. One DCSW participant spoke of continuing to bring a client swimming despite reluctance, as it was advised by a doctor, and the DCSW knew the client usually resisted new things but would grow to enjoy them if given time. Repeated exposure to swimming resulted in the client continuing to go, and enjoying swimming, as the DCSW predicted.

#### Other Training and Techniques to Support Everyday Decision-Making

Eight papers focused on training and techniques other than SDM for adults with intellectual disabilities, nine papers discussed alternate training and techniques for DCSWs, and two papers discussed alternate training or techniques for care partners. One paper named a formal alternative to SDM, Active Support (Beadle Brown et al., 2008; 2012), while the other papers described less formal methods of support which employed a variety of training techniques to facilitate independent decision-making in specific circumstances, such as self-advocacy and personal safety (Daniel et al., 2014; Hickson et al., 2015).

#### Adults with Intellectual Disabilities

Two papers describing training programmes for people with intellectual disabilities were focused on enhancing security and independence in residential settings. Black and colleagues (2009) provided training to support skills development in communicating rights to respect and choice. Johnson and colleagues (2012) provided interviewer skills training to enable participation in support staff selection and recruitment. Participants reported feeling more in control of who supported them in their day-to-day activities, and better able to ensure they were supported effectively. Training programmes described by Hickson et al. (2015) and Daniel et al. (2014) focused specifically on increasing participant awareness of safe decisions to make in unsafe situations, such as assault, or recognising abusive situations. Garcia-Iriarte and colleagues (2009) provided training in how to actively participate in self-advocacy groups through action projects and co-design of support tools.

Three papers detailed techniques for researchers to utilise when involving adults with intellectual disability in research projects. These techniques followed similar suggestions as for DCSWs and care partners; namely, respecting the autonomy of the person with intellectual disability, ensuring they had full understanding of what the research would entail, and building in techniques to facilitate decision-making, such as providing questions ahead of time to give them more time to think or including them as co-researchers (Timmons et al., 2011).

#### Direct Care Support Workers

Nine papers discussed methods used to improve DCSWs’ knowledge of how to support the decision-making of adults with mild to moderate intellectual disability. “Active support”, defined as “a practice whereby staff use an enabling relationship to facilitate the engagement of people with intellectual disabilities in meaningful activities and social relationships” (Bigby et al., 2019a, p. 280), was discussed in two papers by Beadle Brown et al. (2008, 2012). Findings suggested that this approach led to an increase in support quality from staff, resulting in greater engagement from the supported person.

Although adults with more severe intellectual disability were less likely to receive decisional support, four examples of approaches and techniques used to interpret decisions in such cases were included in the review (Calveley, 2012; Murphy et al., 2011; Nicholson et al., 2021; Tracy, 2015). DCSWs and care partners familiar with the adult in question reported being able to interpret bodily cues and behaviour patterns . Body language and repeated interest in activities, foods and people were considered indications of preference and were used to inform care plans for adults with severe intellectual disability.

Two papers described examples of effective and inclusive decision-making practices used by DCSWs. Whitehead and colleagues’ (2016) study of diabetes management demonstrated good practice in decision-making techniques through a client-led system of negotiated autonomy involving clients deciding which aspects of management they wanted help with. Additionally, Hellzen and colleagues (2018) found that encouraging adults with intellectual disability to voice disagreements or displeasures with staff led to feelings of empowerment and better self-advocacy.

#### Care Partners

Training for care partners was discussed in two papers (McCausland et al., 2019; Taylor et al., 2019). Like DCSWs, care partners were encouraged to improve their listening skills, understanding and ability to compromise to support decision-making. One paper, which reported on a pilot programme to help care partners of adults with intellectual disability establish plans for future living and care arrangements, found that formal supports to ensure the adult with intellectual disability could remain at home were often lacking, necessitating a move to residential care (McCausland et al., 2019). Taylor and colleagues (2019) reported that consistency throughout the support circle was considered essential to ensure maximum effective support. However, lack of adequate professional support made facilitation of choice difficult, particularly regarding transitional events, because care partners lacked the resources necessary to support the person with intellectual disability on their own.

## Barriers and facilitators to providing effective support in everyday decision-making

Twenty-seven papers described barriers and facilitators experienced by all three participant groups in relation to support in everyday decision-making. Facilitators identified included having a less institutional living approach (n = 4), respect for the preferences of the person with intellectual disability (n = 5), knowing the person with intellectual disability well (n = 6), having an inclusive, flexible policy in place (n = 4), and having a policy that incorporated support for decision-making (n = 3). Barriers included inflexible policies and practices (n = 3), underestimating and not listening to the person (n = 8), and lack of familiarity with the needs of the person (n = 4).

### Facilitators

In four papers, an open, deinstitutionalised approach to care was associated with greater choice, respect, and decision-making opportunity for adults with intellectual disability in formal care settings (Devi et al., 2020; Haigh et al., 2013; Hollomotz, 2014; Williams and Porter, 2017). Respect for privacy and independence were also crucial to fostering a sense of security, freedom and decisional control for adults with intellectual disability in residential settings (Kahlin et al., 2016). In the family home, involvement of care partners who facilitated adults with intellectual disability in expressing goals and preferences and advocating for their right to have these desires heard had a similar effect (Bigby et al., 2015a; Kahlin et al., 2016; Wass et al., 2021; Williams and Porter, 2017).

### Knowing the Person Well

For adults with intellectual disability, having access to support workers who were committed to vocal advocacy on their behalf with management and familiar with their personal wants, habits, and preferences was vital for supportive decision-making (Antaki et al., 2008; Larkin et al., 2018). Williams and Porter (2017) reported that clients valued a personalised approach to assistance and selected people to help with decisions based on familiarity, trust, and personal investment. For adults with severe to profound intellectual disabilities, having staff that were familiar with their non-verbal forms of communication or resistance was important (Nicholson et al., 2021).

### An Inclusive Policy

Residential homes with inclusive policies were more respectful of residents, who reported feeling heard and included (Beadle Brown et al., 2012; Devi et al., 2020; Petner-Arrey and Copeland, 2015). Khalin and colleagues (2016) found that residential homes who encouraged residents to make decisions about how to personalise semi-private areas were better able to foster a sense of home and decisional control. Hassan demonstrated how residents' person centred plans could be more effective when they incorporated activites they enjoyed (Hassan, 2017). Adults with intellectual disability said they found it easier to understand research if it was explained more slowly (Andre-Barron et al., 2008) and a support person of their choosing was available to help with understanding (Carey and Griffiths, 2017). Trusting the researcher and their information was also important to them (McDonald et al., 2013).

### Barriers

Barriers to effective everyday support in decision-making were seen at all levels of interpersonal and organisational relationships. An important factor identified in three papers was lack of familiarity with the adult with intellectual disability (Charnley et al., 2019; Fisher et al., 2009; Petner-Arrey and Copeland, 2015) which made engaging in effective decisional support more difficult. Factors that increased the likelihood of this occurring included high staff turnover (Petner-Arrey and Copeland., 2015), staff being unavailable to assist (Charnley et al., 2019), the supporter being a temporary professional brought in for decisions (Fisher et al., 2009), lack of organisational level communications (Charnley et al., 2019; Fisher et al., 2009), and direct guardianship practices (Gross et al., 2013; Werner and Chabany, 2016).

### Ineffective Policies

Research examining the application of policies designed to increase independence and facilitate decision-making for adults with intellectual disability found that they often created problems for DCSWs. Antaki and colleagues (2009) found that disability service care policy set unrealistic standards for DCSWs that did not reflect the realities of day-to-day activities. Two studies investigated the decision-making role adults with intellectual disability in independent living situations assigned to organisations and professional carers. They found that the clients still viewed care workers as authority figures rather than supporters or advocates (Pallisera et al., 2021; Fullana et al., 2022).

In three papers, institutional policies regarding safeguarding, paperwork and procedures posed significant barriers to staff in engaging in everyday support in decision-making with clients (Devi et al., 2020; Hollomotz, 2014; Petner-Arrey and Copeland, 2015). Petner-Arrey and Copeland (2015) reported that staff were often underpaid and overworked and were expected to uphold institutional policies even if they felt they infringed on the autonomy and rights of the people they were assisting. Organisational concerns about risk were also a factor, with support workers being uncertain of how to balance the right of their clients to make choices with ensuring their safety (Hollomotz, 2014; Petner-Arrey and Copeland, 2015).

### Care Support Worker Conflicts

DCSWs often met with resistance and conflict during interactions with clients and their care partners when attempting to support their choices (Charnley et al., 2019; Giertz, 2018; Pallisera et al., 2018). Diet was a recurring issue, with care partners wanting DCSWs to prevent residents from eating unhealthy foods and adhere to their own dietary preferences (Cartwright et al., 2015; Gill and Fazil, 2013). Further conflict was reported when care partners did not agree with decisions made by the person with intellectual disability and sought to override them. This was usually based on the premise that care partners knew them best and as such were better suited to make these decisions, and often led to the application of best interest decision-making (Charnley et al., 2019; Fisher et al., 2009; Giertz, 2018).

### Underestimating the Person

A frequent barrier to decisional support was DCSWs’ and care partners’ underestimation of the ability of the adult with intellectual disability to make a decision (Pallisera et al., 2018; Petner-Arrey and Copeland, 2015). Participants with intellectual disability mentioned that they were often treated as if they all liked the same things purely because they had an intellectual disability (Petner-Arrey and Copeland, 2015). They reported that their actual goals and preferences were dismissed by DCSWs and care partners alike, who made incorrect assumptions about their capacity to choose (Charnley et al., 2019; Hoole and Morgan 2011) and felt they were better equipped to make important decisions on their behalf (Ferguson et al., 2011; Gill and Fazil, 2013; Gross et al., 2013; Jingree et al., 2006; Stancliffe et al., 2011; Stefánsdóttir et al., 2018). This was coupled with service concerns about health and safety, which limited residents’ independence in certain tasks. Hollomotz (2014) reported that institutional guidelines often did not consider varying levels of ability in residents and instead employed a strict blanket policy that all residents were required to obey. If these policies were breached, DCSWs were subject to disciplinary action, which residents were aware of. This underestimation and inclination to assume control appeared to be influenced by severity of intellectual disability in some cases (Bigby et al., 2009; Murphy et al., 2011). Bigby and colleagues (2009) found that adults with severe intellectual disability were least likely to be supported to make decisions, with many carers asserting that it was not possible due to their inability to verbally communicate. In their paper discussing transition-aged adults with moderate to profound intellectual disability, Murphy and colleagues (2011) reported that care partners were hesitant to see them as adults, believing they still needed care and guidance.

### Restrictions in Sexual Health Decisions

In eleven papers, adults with intellectual disability reported that they were often not permitted to make decisions about their sexual health. For women with intellectual disability, the question of pregnancy and reproductive health arose often as a source of worry and complication (McCarthy, 2010; Engwall, 2014). Two studies found that healthcare professionals were unused to engaging with patients with intellectual disability and frequently failed to include them in decision-making regarding their health. Accompanying DCSWs often compounded this by preventing their involvement during doctor visits (Ferguson et al., 2011; McCarthy, 2010).

Pregnancy was a source of concern for both DCSWs (McCarthy, 2010) and care partners (Jamieson et al., 2016), and often a source of fear for women with intellectual disability themselves (Engwall, 2014). Engwall (2014) reported women with intellectual disability felt unable to have children because of their disability. Fear of disclosure of pregnancy due to a lack of openness with family members (Ledger et al., 2016), a tendency for parental values to influence their views of pregnancy as something unacceptable or dangerous (McCarthy, 2010), and fears of losing services and supports (Jamieson et al., 2016) were also reported.

Bigby and colleagues’ 2009 paper discussing adults with severe intellectual disability and choice making reported that DCSWs viewed masturbation or other forms of sexual expression by residents as “that dirty thing” or “inappropriate behaviour” (p. 367), reinforcing the notion that adults with intellectual disability are not adults, with the same freedom to express their decisions around sexuality as most adults without intellectual disability do. This was highlighted starkly by Roets and colleagues (2006), who provided a detailed account of the attempts of a young woman with intellectual disability to resist efforts to force her to have a hysterectomy due to fears she would become pregnant. The woman was supported by her Advocate (an official paid role in her native Belgium) to prevent this from occurring.

## Discussion

Support in everyday decision-making has become an increasingly important topic amongst carers and policymakers. Our findings indicate that adults with intellectual disability, their care partners and DCSWs have complex and often overlapping perceptions and experiences of support in everyday decision-making, adopt a variety of techniques and approaches to facilitate it, and encounter a range of barriers and facilitators in its implementation. Support in everyday decision-making is widely recognised as important, but there is a lack of consistency in the literature regarding its description and operationalisation. Although sometimes described in a supportive manner that respects the will and preference of the person, at other times it appears purely paternalistic in execution (Fisher et al., 2009; Scholten et al., 2021). Difficulties are particularly pronounced when the person with intellectual disability has higher support needs and/or is nonverbal (Bigby et al., 2009; Calveley, 2012). Researchers can address this by working directly with adults with intellectual disability, their care partners and DCSWs to streamline the language and methodologies of support in everyday decision-making.

Our review identified a paucity of research examining specific methods of support. In 70 papers, decision-making was explored in the broader context of choice and control within the family home, encouragement (or lack thereof) of independence by support workers in residential care, or self-advocacy in the daily lives of people with intellectual disability, all factors that indirectly contribute to decisional support. This lack of focus on specific support could be rectified by encouraging adults with intellectual disability, their care partners and DCSWs to consciously integrate a method such as SDM into their daily lives through education and training. SDM was identified as the most widely used method of support in 11 papers, three of which focused on the LaTrobe Framework, a training initiative specifically designed to help stakeholders improve their ability to use SDM in their lives (Bigby et al., 2022a, 2022b; Carney et al., 2021). This framework’s approach to the implementation of SDM through training, education, and stakeholder inclusion is one that facilitates engagement with SDM as a concept and brings approaches of supporters in line with the CRPD. Many of the findings echo those seen in the literature on support in everyday decision-making in people with cognitive impairments such as dementia or TBI (Bigby et al., 2015b; Kohn and Blumenthal, 2014; Noorlandt et al., 2020). Having the support of known and trusted people in making decisions, identified as being central to positive support experiences among adults with TBI and dementia (Bigby et al., 2015b; Kohn and Blumenthal, 2014; Noorlandt et al., 2020), also emerged as a key factor for people with intellectual disability (Andre-Barron et al., 2008; Carey and Griffiths, 2017; McDonald et al., 2013) and their carers (Bigby et al., 2015a; Burke et al., 2019) in this review. Similarly, the finding that DCSWs acted as a bridge between adults with intellectual disability and their families, attempting to balance the wishes and desires of both parties while also adhering to service policy requirements (Andre-Barron et al., 2008; Bigby et al., 2011, 2019b), has been observed in research involving people with cognitive impairments such as dementia (Daly et al., 2018; Donnelly, 2019; Dukes and McGuire, 2009; Mansell and Beadle‐Brown, 2004).

DCSWs’ engagement in practices to support everyday decision-making seemed to largely depend on the policies of the service they worked for, and training opportunities were limited (Devi et al., 2020; Hollomotz, 2014; Petner-Arrey and Copeland, 2015). This has been seen elsewhere, as service policy is a frequently identified barrier to decisional freedom for people with TBI, mental health conditions and dementia (March et al., 2013; O’Hara, 2008; Davidson et al., 2015). Active support shows promise as a support approach (Beadle Brown et al., 2008, 2012; Mansell and Beadle Brown, 2012). However, it has been mainly studied in residential settings with an emphasis on theoretical application. Recent studies indicate its potential application in the community ((Bigby and Anderson, 2021; Bigby and Wiesel, 2015), but more research is required to examine its applicability to other care service settings and differing levels of intellectual disability.

Ultimately, it can be concluded from the literature that support in everyday decision-making requires inclusive and respectful support of the person with intellectual disability by their carers (Nonnemacher and Bambara, 2011; Whitehead et al., 2016). The findings of this review support the view that decision-making methods such as substitute or best interest decision-making are a barrier to effective support (Charnley et al., 2019; Fisher et al., 2009; Giertz, 2018). Whether these methods were used appeared to be dictated by severity of intellectual disability, with the preferences of adults with more severe intellectual disability frequently being overlooked and underestimated (Bigby, 2008; Murphy et al., 2011). This assumption also appears to hold for people who have experienced severe TBI (Bigby et al., 2015a; 2015b). People with more severe intellectual disabilities were significantly underrepresented in the papers included in the present review and rarely actively participated even when included. Greater inclusion of adults with severe to profound intellectual disability in making decisions about their lives could be achieved using tailored approaches such as that described by Nicholson and colleagues (2021), or via communication aids (Stewart et al., 2018) or choice making technologies (Davies et al., 2003).

## Implications for further research

Although the research summarised in this review offers insights into the experiences and perceptions of support in everyday decision-making, there are clear gaps in the literature that need to be addressed. Firstly, many of the findings regarding everyday decision-making were negatively oriented (Charnley et al., 2019; Fisher et al., 2009; Hollomotz, 2014; Petner-Arrey and Copeland, 2015), with research focusing on barriers to decisional support. Few of the included papers set out to identify successful approaches or factors that may help to facilitate decisional support (Bigby et al., 2015b; Burke et al., 2019). It is important that research seeks strategies to resolve barriers, not merely highlight them. Further research is needed to identify what can be done to improve negative experiences and to explore positive and successful experiences of decisional support. Furthermore, it should be acknowledged that many adults with intellectual disabilities can and do make decisions independently or with minimal assistance. It is important that future research highlights this to avoid paternalistic application of decisional support through presumption of its necessity for every person with an intellectual disability and/or in all instances of decision-making.

It is important to include all stakeholders involved in decisional support in research in order to identify what they need and to improve everyday decision-making practices. The lack of cohesion between the views and experiences of the three groups is another factor that future research must address. Most papers in this review included only one or two of the stakeholder groups, with only ten papers having representation from all three groups. McCausland and colleagues (2019) also raised a vital point- namely, decisions such as whether to remain at home or move to residential care are heavily affected by available resources. This suggests that further government investment and community outreach may be more important than individual- or family-level interventions for future planning to be successful. Future research should continue to address this issue by focusing on improving policy and resources to ensure adults with intellectual disabilities have the means to live where they choose. Finally, the lack of formal, clearly delineated decisional support techniques or approaches in the included papers represents a distinct gap in the literature. The LaTrobe framework provides training for supporters to help them develop skills in supporting adults with intellectual disability using SDM as a technique, and has promising results (Douglas and Bigby, 2020). Some theoretical data on the approach of the National Resource Centre for Supported Decision Making in the US (NRCSD: Blanck and Martinis, 2015) has been published, but is very specific to the legal context of the individual US state it is focused on. Further research is needed on this approach, and consideration of its implementation and scalability in countries with differing care structures. Finally, few papers involving self-advocacy groups were included in this review. It is vital for research going forward to highlight the work and voices of self-advocates to ensure the centrality of people with intellectual disabilities (Deguara et al., 2012; Gilmartin and Slevin, 2010; McCausland et al., 2018).

### Strengths and limitations

This was the first review to focus exclusively on everyday decision-making in adults with intellectual disabilities and their supporters. The comprehensive search capturing the changing terminology within this research field is a key strength of this review. In addition, the inclusion of research involving adults with intellectual disabilities, as well as their families and professional carers allowed their different viewpoints to be represented, giving a more complex and nuanced overview of support in everyday decision-making. Most included papers involved adults with intellectual disability as active, central participants and ensured their experiences were central within the data.

It is also important to note that the decisions described in these studies were personal decisions for the individuals, and that other kinds of decisions, such as interpersonal decisions were not included. Further research is needed to determine the place of SDM in interpersonal resolution of decisional outcomes and conflict among included decision-makers.

The review was limited by only including papers written in English, with most emanating from the UK, Australia, and the USA; data may be omitted from other nations where different approaches to decisional support may have been adopted.

## Conclusion

Support in everyday decision-making is a rapidly growing area of interest that is still ill-defined and under-investigated. Many studies allude to this practice without directly addressing how it is best executed. This lack of specificity indicates the need for further research on this important topic. SDM has emerged as the most widely used and investigated approach to providing support in this context, but questions remain as to how it can be applied most effectively. It is vital that adults with intellectual disability, their care partners and DCSWs are central to discussions surrounding support in everyday decision-making, to allow for the formation of systems that acknowledge and address their needs and fit their perceptions of what is required. These groups are often addressed separately in research, even though decision-making for adults with intellectual disability is usually a collaborative process; the integration of their viewpoints is thus of utmost importance to allow for a more rounded, nuanced view of the topic, and the identification of an approach that works best for all involved. Furthermore, it is important to ensure adults with intellectual disabilities have a central role in research on SDM; more emphasis should be placed on self-advocacy and developing processes and structures that ensure meaningful opportunities for engagement across the research lifecycle.

## Supplemental Material

Supplemental Material - Experiences and perceptions of everyday decision-making in the lives of adults with intellectual disabilities, their care partners and direct care support workers Supplemental Material for Experiences and perceptions of everyday decision-making in the lives of adults with intellectual disabilities, their care partners and direct care support workers by Hannah Casey, Áine Trayer, Deirdre Desmond and Laura Coffey in Journal of Intellectual Disabilities

Supplemental Material - Experiences and perceptions of everyday decision making in the lives of adults with intellectual disabilities, their care partners and direct care support workers Supplemental Material for Experiences and perceptions of everyday decision making in the lives of adults with intellectual disabilities, their care partners and direct care support workers by Hannah Casey, Áine Trayer, Deirdre Desmond and Laura Coffey in Journal of Intellectual Disabilities
